# Semi-standardized evaluation of extraprostatic extension and seminal vesicle invasion with [^18^F]PSMA-1007 PET/CT: a comparison to MRI using histopathology as reference

**DOI:** 10.1186/s41824-024-00234-4

**Published:** 2025-01-03

**Authors:** Erland Hvittfeldt, Fredrik Hedeer, Erik Thimansson, Kevin Sandeman, David Minarik, Jacob Ingvar, Anders Bjartell, Elin Trägårdh

**Affiliations:** 1 Department of Translational Medicine, Lund University, Lund, Sweden; 2 Department of Clinical Physiology and Nuclear Medicine, Skåne University Hospital, Malmö, Lund, Sweden; 3 Wallenberg Centre of Molecular Medicine, Lund University, Lund, Sweden; 4 Department of Radiology, Helsingborg Hospital, Helsingborg, Sweden; 5 Department of Pathology, Region Skåne, Malmö, Sweden; 6 Department of Urology, Skåne University Hospital, Malmö, Sweden; 7 Radiation Physics, Skåne University Hospital, Malmö, Sweden

**Keywords:** PSMA PET/CT, MRI, Prostate cancer, T staging

## Abstract

**Background:**

Positron emission tomography/computed tomography (PET/CT) with prostate specific membrane antigen ligands (PSMA) is established for use in primary staging of prostate cancer to screen for metastases. It has also shown promise in local tumor staging, including detection of extraprostatic extension (EPE) and seminal vesicle invasion (SVI). Previous studies have shown high heterogeneity in methods and results. Our aim was to compare [^18^F]PSMA-1007 PET/CT to magnetic resonance imaging (MRI) in evaluation of EPE and SVI, building on a previously described method for standardized evaluation. We retrospectively included 124 patients who had undergone MRI, PSMA PET/CT and prostatectomy. PSMA PET/CT images were evaluated by two nuclear medicine physicians. Using a standardized method, they measured length of capsular contact (LCC) and assessed EPE and SVI visually with the use of 5-point Likert scales. A radiologist evaluated MRI images using criteria based on Prostate Imaging–Reporting and Data System version and incorporating LCC measurement and Likert scales. We evaluated diagnostic performance with histopathology as reference, and the interrater reliability of the PET evaluations.

**Results:**

The sensitivity and specificity for detecting EPE with the quantitative LCC method for PSMA PET/CT was 0.46/0.91, for the visual method 0.28/0.82 and for the combination of the two 0.54/0.76. AUC in ROC analysis for the LCC method was 0.70. For MRI the sensitivity and specificity were 0.80/0.64. For SVI, PET/CT and MRI had sensitivity and specificity of 0.14/1.0 and 0.50/0.92 respectively. The intraclass correlation coefficient for the PET LCC measurement was 0.68, the kappa values for the visual Likert scales for PET were 0.53 for EPE and 0.63 for SVI.

**Conclusions:**

In this study, we attempted to standardize quantitative and qualitative PSMA PET/CT evaluation of EPE and SVI and compare the method with MRI. MRI had a higher sensitivity for EPE while PSMA had a higher specificity. For SVI, both methods had high specificity. The interrater reliability for the PSMA PET/CT evaluations was moderate to substantial.

**Supplementary Information:**

The online version contains supplementary material available at 10.1186/s41824-024-00234-4.

## Introduction

Prostate cancer (PC) is the second most common form of cancer worldwide (Bray et al. 2024). When classifying (PC), the distinction between organ-confined disease and cancer with an extraprostatic component is an important prognostic factor (Mottet et al. 2021). In the TNM (tumour, node, metastasis) classification this corresponds to T1–T2 and T3–T4 stages, respectively. T3 is subdivided into T3a (extraprostatic extension, EPE) and T3b (seminal vesicle invasion, SVI) while T4 represents tumor invading adjacent structures, such as the rectum or urinary bladder (Buyyounouski et al. 2017). Identifying patients with EPE or SVI influences risk stratification and treatment planning, notably the choice between radical surgery or radiation therapy and the selection of patients suitable for nerve-sparing surgery (Mottet et al. 2021; Eastham et al. 2022).

The role of imaging in T3 staging is unclear. The TNM classification states that clinical T-stage should be based on digital rectal exam only, and this is reflected in both European and American guidelines (Mottet et al. 2021; Buyyounouski et al. 2017; Eastham et al. 2022). However, all three materials acknowledge that magnetic resonance imaging (MRI) evaluated by an experienced radiologist can provide additional information on the extent of local disease. This information may be used in treatment planning, especially in high-risk disease. Prostate-specific membrane antigen positron emission tomography (PSMA/PET) with computed tomography (PET/CT) or MRI (PET/MRI) has become an important modality for imaging of PC, but mainly for N- and M-staging (local lymph nodes and distant metastases) in high-risk disease and in biochemical recurrence after curative treatment. Current guidelines do not recommend it for T-staging (Mottet et al. 2021; Eastham et al. 2022; Lowrance et al. 2023).

Several studies have evaluated T-staging using PSMA PET. A 2020 meta-analysis of 12 studies assessing T-staging with PSMA PET (in combination with CT or MRI) reported a pooled sensitivity and specificity of 0.72/0.87 for EPE and 0.68/0.94 for SVI (Woo et al. 2020). A more recent meta-analysis focusing only on head-to-head studies comparing PSMA PET to MRI (9 studies) found pooled sensitivity and specificity for PSMA PET of 0.59/0.79 for EPE and 0.51/0.93 for SVI, compared to MRI values of 0.66/0.76 for EPE and 0.60/0.96 for SVI (Ma et al. 2024). For all modalities the individual studies showed highly variable performance. This variability was underscored in a 2020 review which emphasized the need for standardized imaging techniques and reporting, particularly for PSMA PET (Abrams-Pompe et al. 2021). While MRI evaluation and reporting have been standardized with PI-RADS, reporting standards for PSMA PET are less detailed, especially for T-staging (Ceci et al. 2021; Turkbey et al. 2019). The measurement of the length of capsular contact as an indirect indication of the risk of T3a has been studied for MRI, showing diagnostic performance comparable to, and interrater reliability surpassing, that of subjective analysis (Rosenkrantz et al. 2016; Kim et al. 2020). Few studies have explored LCC for PSMA PET, but Brauchli et al. introduced a semi-standardized method for measuring LCC, providing a potential framework for further research (Brauchli et al. 2020).

Our primary aim in this study was to evaluate the diagnostic performance of [^18^F]PSMA-1007 PET/CT in T3 staging and compare it to MRI, using histopathology as reference method. In addition to the semi-standardized measurement of LCC from Brauchli et al., we used 5-point Likert scales for visual evaluation of EPE and SVI. We also evaluated the interrater reliability of the PSMA PET/CT evaluations.

## Methods

### Subjects

We retrospectively included patients with newly diagnosed, biopsy-verified prostate cancer who underwent radical prostatectomy at Skåne University hospital between September 2019 and March 2023, and who had performed both PSMA PET/CT and MRI before surgery. A maximum of 180 days between the first imaging procedure and surgery was accepted. From September 2019 to February 2021 intermediate- and high-risk prostate cancer patients according to D’Amico (PSA ≥ 10 or Gleason score ≥ 7 or clinical T stage ≥ T2b (D'Amico et al. 1998)) were accepted for PSMA PET/CT. From February 2021 to March 2023 only high-risk patients were accepted (PSA ≥ 20 or Gleason score ≥ 8 or clinical T stage ≥ T2c).

### Imaging

#### PSMA PET/CT imaging

Patients were injected with 4.0 MBq/kg of [^18^F]PSMA-1007 and imaging was performed after 120 min. Head to knee PET scans were performed on one of four GE Discovery MI PET/CT systems (GE Healthcare, Milwaukee, USA) at two nuclear medicine sites (Skåne University hospital, Lund and Malmö, Sweden), all using the same protocol. The acquisition time was 2 min/bed position (3 min for BMI > 40). The Q.-Clear reconstruction algorithm (GE Healthcare, Milwaukee, USA) was used, including time-of-flight, point spread function and CT-based attenuation correction with a 256 × 256 matrix (pixel size 2.7 × 2.7 mm2, slice thickness 2.8 mm). The noise regularization parameter was set to 800. The CT was of diagnostic quality (100 kV/80–480 mA), and intravenous and oral iodine contrast agents were administered unless contraindicated (in two patients). An adaptive statistical iterative reconstruction technique was used to reconstruct the CT images in a 512 × 512 matrix, slice thickness 5 mm. The fused images were viewed with Hermes Hybrid Viewer 6.1.3 (HERMES medical solutions, Stockholm, Sweden).

#### MRI imaging

MRI scans were performed at eight different radiology sites in the Swedish county Region Skåne. Seven varieties of MRI scanner with field strengths of 1.5 and 3 T were used. Local protocols varied but all included transversal, coronal, and sagittal T2-weighted (T2w) turbo spin-echo images and diffusion weighted transversal images, in compliance with the Prostate Imaging–Reporting and Data System (PI-RADS) version 2 (Weinreb et al. 2016).

### Image analysis

All analyses were performed independently and blinded to patient data. The prostate was divided into 12 segments along left/right, ventral/dorsal, and base/mid/apex lines. In the initial analyses all lesions in contact with the prostate capsule were noted and analyzed as described below. When comparing modalities, lesions were considered matching when they included the same or directly neighboring sections. For SVI only the side was noted. See also supplementary material Fig. 1.

**Fig. 1:**
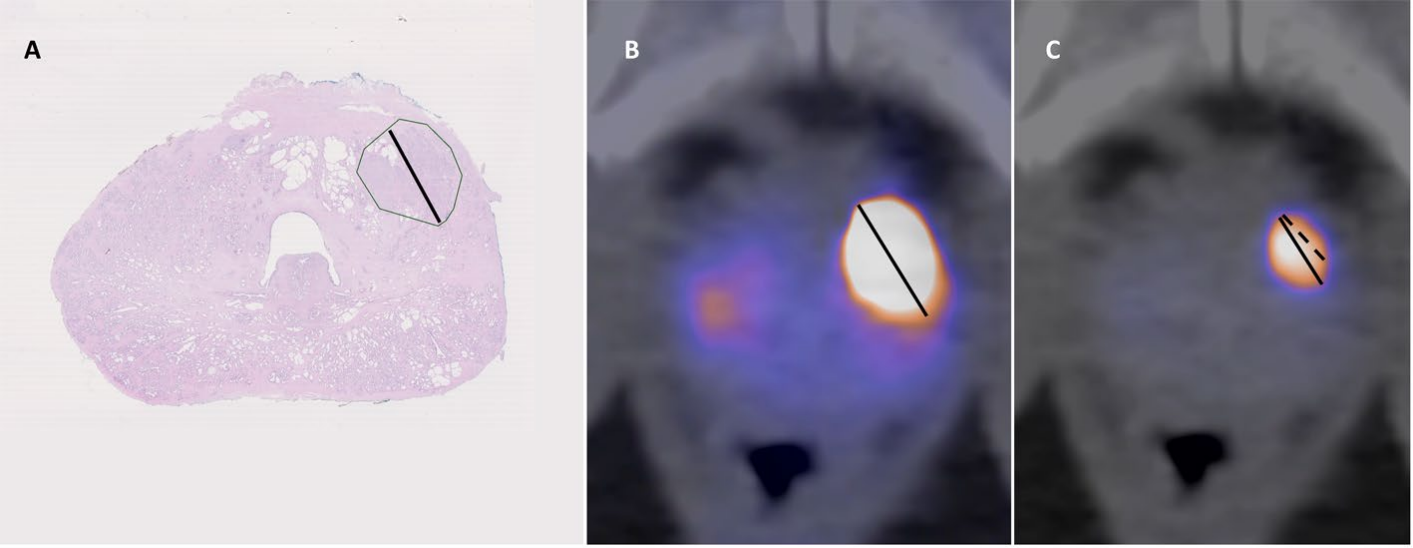
**A** Histopathology shows prostate cancer (green outline) with Gleason 4 + 4 = 8 in the left ventral apical peripheral zone, largest diameter 10 mm (black line). No EPE was recorded. **B** PSMA PET/CT shows intense uptake with a SUVmax of 56. The diameter is measured to 17 mm with max window level set to 10. **C** With window level set to 56 the diameter is measured to 11 mm. Both reviewers measured LCC to 10 mm (dashed line)

#### PSMA PET/CT analysis

**Fig. 2:**
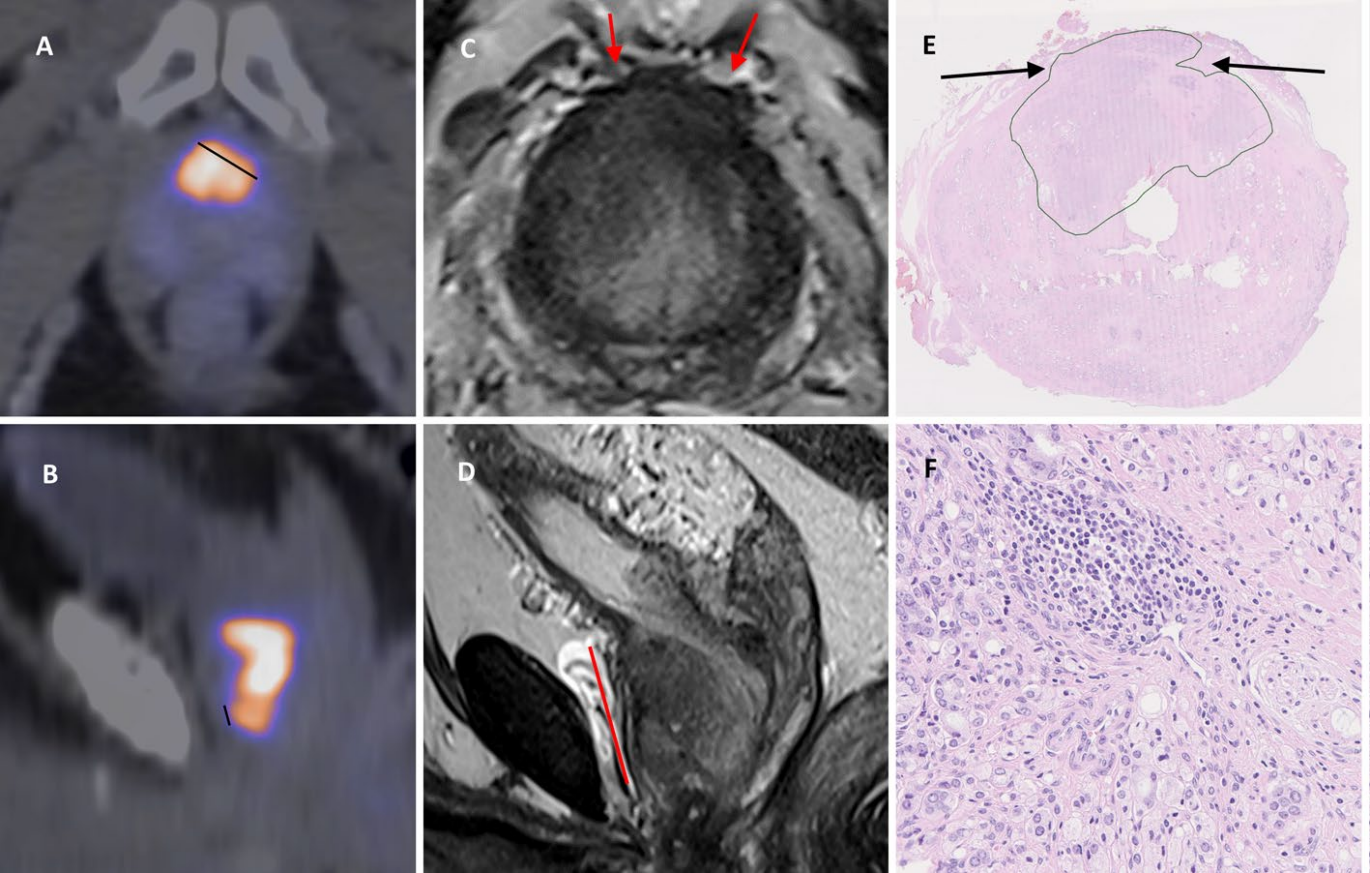
**A**, **B** PSMA PET/CT shows intense heterogenous uptake ventrally in the prostate. SUVmax was 47.5 but measurements were also made with max window level 39.7 due to heterogeneity. Reviewer 1 measured maximum LCC to 16 mm in the axial plane (**A**, black line), reviewer 2 to 5 mm in the sagittal plane (**B**). Both reviewers set visual Likert to 2. **C**, **D** MRI shows a ventral PI-RADS 5 lesion with indirect signs of EPE (bulging contour; red arrows in axial plane T2W in **C**). LCC 30 mm in sagittal T2w images in **D**. **E**, **F** Histopathology shows a ventral Gleason 4 + 5 lesion (**F**) in the mid prostate (**E**, green outline) with EPE up to 14 mm in length and 1.5 mm in depth (**E**, arrows). EPE extends ventrally in a sagittal direction approximately 20 mm towards the prostate base

One nuclear medicine specialist (FH, 15 years’ experience with PET, 6 years with PSMA) and one nuclear medicine resident (EH, 6 years’ experience with PET, 4 years with PSMA) analyzed the images. For each lesion visually deemed as probable PC the LCC was measured using the method described by Brauchli et al., and with the same color look up table (LUT) (NEMA hot metal blue) (Brauchli et al. 2020). To summarize the method, the upper SUV window level was set to the SUVmax of the lesion in question. In heterogenous lesions where the lesion SUVmax was distant from the prostate capsule, the reviewer measured SUVmax closer to the capsule (Figs. 1 and 2). The length of the capsular contact (LCC) was measured as the longest straight line between two points on the tumor-capsule interface in the axial, sagittal or coronal orientation. In addition to the LCC measurement, the lesion was visually assessed (with the same window levels) for EPE and SVI using 5-point Likert scales (Table 1). For synchronization between raters ten patients not included in the study were jointly analyzed.

**Table 1:** Instructions for Likert scale for PSMA PET

Likert	EPE	SVI
1. EPE/SVI highly unlikely	No capsular contact	No vesicle contact
2. EPE/SVI unlikely	Capsular contact but no uptake outside the prostate	Vesicle contact but no uptake in the vesicle
3. EPE/SVI possible	Equivocal–capsular contact but no definite suspicion of uptake outside the prostate	Equivocal–vesicle contact but no definite suspicion of uptake in the vesicle
4. Suspected EPE/SVI	Suspicion of pathological uptake outside of the prostate	Suspicion of pathological uptake in the vesicle
5. EPE/SVI highly likely	Strong suspicion of pathological uptake outside of the prostate	Strong suspicion of pathological uptake in the vesicle

A composite PET evaluation was created for all lesions considered probable PC by both readers. The composite LCC was the mean of the readers’ measurements. A composite visual evaluation of EPE or SVI was created from the 5-point Likert scale. Likert scores of 1–2 were considered negative, 3 equivocal, and 4–5 positive. When both readers were positive, negative or equivocal this was used as the composite. If one reader was equivocal and the other positive or negative the composite was set to the positive or negative. If one reader was positive and the other negative an agreement was reached through a mutual second look at the images.

#### MRI analysis

A specialist in radiology (ETh) with 8 years’ experience of prostate MRI reviewed the images using Sectra IDS7 PACS (Sectra Medical, Linköping, Sweden). 5 point Likert scales recommended by the Swedish national clinical cancer care guidelines were used for assessing EPE and SVI (Regionala cancercentrum 2024). The EPE Likert scales for MRI incorporate an LCC measurement with a cut-off of 12 mm (Table 2).

**Table 2:** Instructions for Likert scales for MRI

Likert	EPE	SVI
1. EPE/SVI highly unlikely	No capsular contact	Not defined
2. EPE/SVI unlikely	Capsular contact < 12 mm, no signs of EPE	Not defined
3. EPE/SVI possible	Capsular contact ≥ 12 mm, no signs of EPE *or* capsular contact < 12 mm with indirect signs*	Tumor has contact with SV entry point in the prostate
4. Suspected EPE/SVI	Capsular contact ≥ 12 mm with indirect signs of EPE	Thickening of lower SV wall with restricted diffusion
5. EPE/SVI highly likely	Measurable radial extraprostatic component	Low signal, restricted diffusion tumor in the SV base

*Bulging prostatic contour, irregular or spiculated margin, thickened or broken capsular line

### Histopathological evaluation

In addition to the routine clinical evaluation of prostatectomy specimens, a second evaluation was performed by a pathology specialist with 10 years’ experience of prostate cancer pathology. All slides from the prostatectomy specimens were annotated and evaluated using the digital pathology system Sectra Digital Pathology solution (Sectra Medical, Linköping, Sweden). The length, depth, and location of EPE was noted for all T3a lesions. For T3b lesions the side of SVI was noted.

### Statistical analysis

#### Diagnostic performance

We calculated the sensitivity, specificity, accuracy ([TP + TN]/n), positive predictive value (PPV) and negative predictive value (NPV) for MRI and PSMA PET/CT with histopathology as gold standard. For these diagnostic performance indices < 0.70 was considered low, 0.70–0.90 moderate and > 0.90 high. The calculations were made both on a per patient and per lesion level, and excluding patients where no pathological lesions were identified. Calculations were made with equivocal cases (Likert 3) counted as positive and as negative. In addition to the 10 mm LCC cut-off suggested by Brauchli et al. we determined the optimal cut-off in our material by calculating Youden’s index (sensitivy + specificity-1).

#### Interrater reliability

For interrater reliability (IRR) calculations for the PSMA PET/CT Likert scales, we used Cohen’s weighted kappa (k) with linear weights. For the LCC measurements, we used Intraclass correlation coefficient (ICC) (2-way random, absolute agreement, single measures). Agreement was considered poor for values < 0.20, fair for 0.21–0.40, moderate for 0.41–0.60, substantial for 0.61–0.80 and near perfect for > 0.80.

The statistical analyses were made with IBM SPSS Statistics 29 and Epitools (for exact calculation of CI:s when any performance measurement was zero) (Sergeant 2018).

## Results

### Subjects

We identified 965 patients who performed PSMA PET/CT and MRI in the chosen time period. Prostatectomy had been performed on 172 of these patients. In 145 of these patients less than 180 days had passed between first imaging and surgery. 124 of these patients signed informed consent for inclusion. Subject characteristics are in Table 3.

**Table 3:** Subject characteristics

Age (years)	64 (42–78)
PSA (µg/L)	12.2 (1.2–96)
Clinical T-stage from digital rectal exam (n)	
cT1	34
cT2	70
cT3	20
Time between MRI and PET (days)	52 (1–150)
Time between imaging and surgery (days)	58 (1–126)
ISUP grade from needle-core biopsy (n)	
1	2
2	18
3	27
4	40
5	37
D’Amico risk classification (n)	
High	94
Intermediate	30

Values are mean (range)

### Imaging and histopathology

PSMA PET/CT identified 186 tumor lesions, MRI 123 and pathology 169. For 6 PET lesions a mutual second look was needed for agreement on EPE. All these lesions were decided on as equivocal. When comparisons of EPE with histology were made on a per lesion basis, 169 comparisons were made for PET and 168 for MRI (see also supplementary material Fig. 1B). The prevalence in patients of histopathology-verified EPE and SVI was 40% (n = 50) and 11% (n = 14).

### Diagnostic performance

**Fig. 3:**
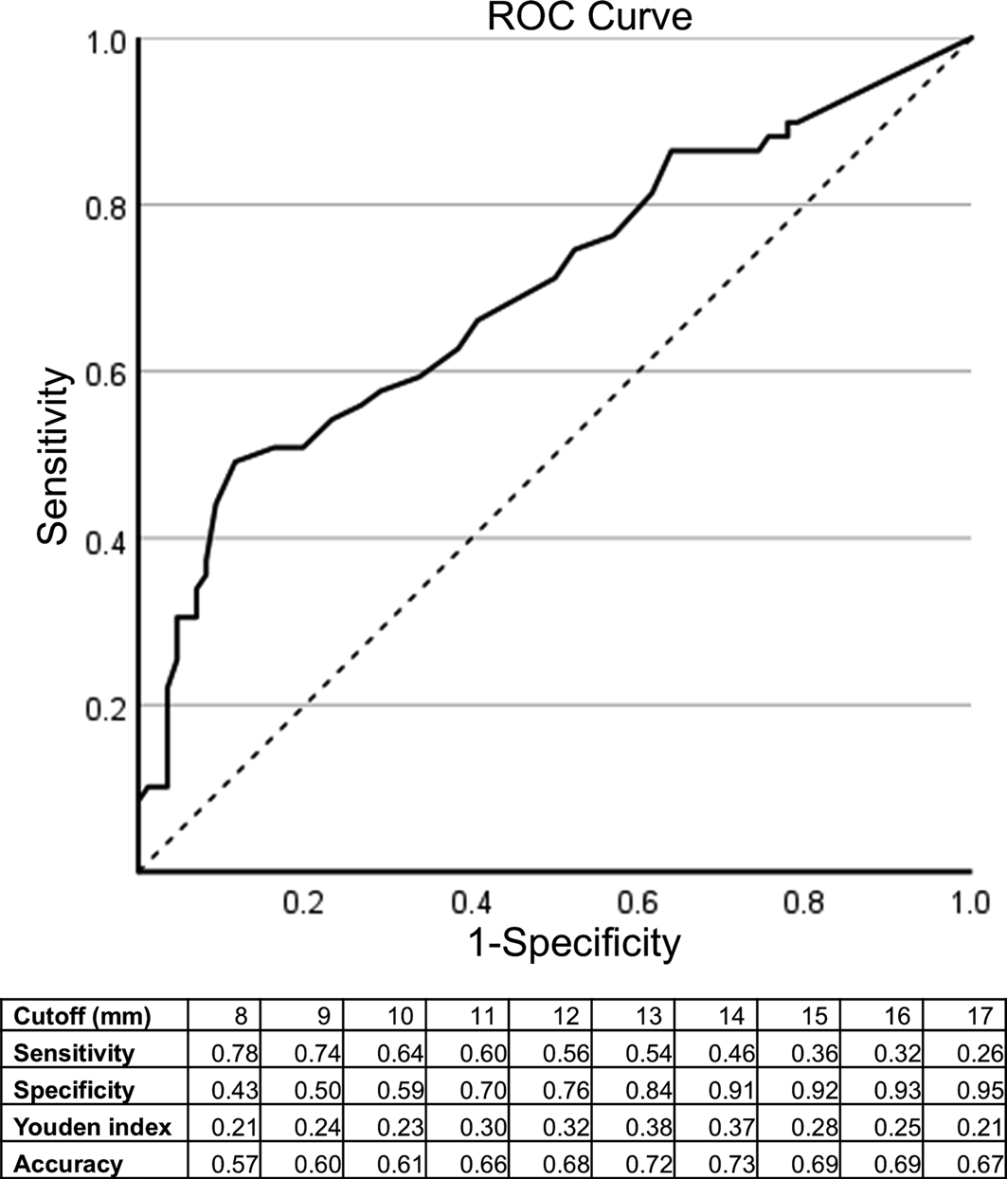
ROC curve for the PSMA PET/CT LCC parameter, with table showing diagnostic indices for different LCC cut-offs. Area under the curve is 0.70 (95% CI 0.61–0.79)

Sensitivity, specificity, accuracy and contingency tables for PSMA PET/CT and MRI are presented in Tables 4 and 5. These analyses are on a per patient basis, consider Likert 1–3 negative and 4–5 positive, and with a PET LCC cut-off of 10 mm unless otherwise noted. ROC curve for the PET LCC parameter is presented in Fig. 3, AUC was 0.70 (95% CI 0.61–0.79). Maximum Youden’s index (0.37–0.38) was for a 13–14 mm cut-off. Additional analyses, including PPV, NPV and all contingency tables, are available in supplementary Table 1.

**Table 4:** Diagnostic performance indices

	Sensitivity	Specificity	Accuracy
EPE PET combined	0.68 (0.54–0.80)	0.54 (0.32–0.65)	0.60 (0.51–0.68)
EPE PET combined*	0.54 (0.40–0.67)	0.76 (0.65–0.85)	0.67 (0.59–0.75)
EPE PET visual	0.28 (0.17–0.41)	0.82 (0.73–0.90)	0.60 (0.52–0.69)
EPE PET LCC	0.64 (0.50–0.76)	0.60 (0.48–0.70)	0.61 (0.53–0.70)
EPE PET LCC*	0.46 (0.33–0.60)	0.91 (0.83–0.96)	0.73 (0.65–0.80)
EPE MRI	0.80 (0.68–0.89)	0.64 (0.52–0.74)	0.70 (0.62–0.78)
SVI PET	0.14 (0.03–0.38)	1.0 (.97–1.0)	0.90 (0.85–0.96)
SVI MRI	0.50 (0.26–0.75)	0.92 (0.86–0.96)	0.87 (0.81–0.93)

All analyses are on a patient basis (n = 124). Equivocal (Likert 3) counts as negative. LCC ≥ 10 positive for PET except* where LCC ≥ 14 positive. Combined PET result positive if either visual or LCC result positive. 95% CI in parenthesis

**Table 5:** Contingency tables

	EPE histology positive	EPE histology negative	Totals for PET or MRI
EPE PET combined			
Positive	34	34	68
Negative	16	40	56
EPE PET visual			
Positive	14	13	27
Negative	36	61	97
EPE PET LCC			
Positive	32	30	62
Negative	18	44	62
MRI EPE			
Positive	40	27	67
Negative	10	47	57
Totals for EPE histology	50 (prevalence 40%)	74	

	SVI histology positive	SVI histology negative	
PET SVI			
Positive	2	0	2
Negative	12	110	122
MRI SVI			
Positive	7	9	16
Negative	7	101	108
Totals for SVI histology	14 (prevalence 11%)	120	

All analyses are on a patient basis (n = 124). Equivocal (Likert 3) counts as negative. LCC ≥ 10 positive. Combined PET result positive if either visual or LCC result positive

### Interrater reliability

The ICC for the LCC measurement was 0.68 (95% CI 0.41–0.82). The weighted k for the visual Likert scale for EPE was 0.53 (0.43–0.63) and for SVI 0.63 (0.46–0.80).

## Discussion

### Diagnostic performance and comparisons with literature

In the present study we evaluated the diagnostic performance of PSMA PET/CT and MRI in T3 staging of prostate cancer. For EPE with PSMA PET/CT, on a patient basis, a moderate accuracy of 0.73 was achieved using only the LCC evaluation with a cut-off of 14 mm, with a high specificity of 0.91 but low sensitivity of 0.46. The visual and combined visual and LCC methods had lower accuracies between 0.60–0.70. The visual evaluation had moderate (0.82) specificity but very low (0.28) sensitivity. MRI had moderate accuracy (0.70), with moderate sensitivity (0.80) and low specificity (0.64). For SVI both PSMA PET/CT and MRI had high specificity (> 0.90) with low sensitivity. The low prevalence (11%) of SVI makes these numbers uncertain. Note that accuracy, while convenient for comparisons, should be interpreted with care—it is highly dependent on prevalence. Depending on the clinical situation a lower accuracy may be acceptable to achieve higher sensitivity or specificity. On a lesion basis both PSMA PET/CT and MRI showed similar sensitivities but higher specificities, mainly due to more true negatives which make little difference clinically (Supplementary Table 1).

Previous studies have shown highly variable results for T3 staging with PSMA PET/CT. For example, a recent meta-analysis reported ranges of accuracy to 0.53–0.90 for EPE (7 studies) and 0.61–0.98 for SVI (9 studies), with pooled accuracies of 0.73 and 0.87 respectively (Gossili et al. 2023). Among these in total 9 studies, 6 did not report how EPE or SVI was evaluated other than “visually”. Two studies used the MRI criteria “angulated contour of the prostate gland or obliteration of the rectoprostatic angle” for EPE. One study used the similar “irregular prostate outline or involvement of extraprostatic structure”. These may not be applicable to PET images which do not directly visualize the contour or outline of the prostate. A recent large (n = 600) multicenter study (Donswijk et al.) with different PSMA tracers found sensitivity/specificity for EPE and SVI to be 0.58/0.59 and 0.30/0.97 respectively (Donswijk et al. 2024). ROC curve AUC was 0.59 for EPE. The study used a 3-point Likert scale with consensus-based criteria focused on tracer uptake outside the prostate and morphological CT findings. None of the studies in the meta-analysis discussed LUTs or window levels.

In this study LCC performed better than the visual evaluation but cases with evident EPE on imaging likely have not been included (see below under Limitations). This may lead to an underestimation of the performance of the visual method compared to the LCC method. Therefore, we believe that visual evaluation should form part of T3 staging with PSMA PET/CT unless prospective studies show otherwise.

Brauchli et al. achieved sensitivity and specificity of 0.74 and 0.86 for EPE with PSMA PET/CT using the same LCC method as in our study, compared to ours of 0.64 and 0.60 with their suggested cut-off of 10 mm (Brauchli et al. 2020). Their study used [^18^F]DCFPyL which, like most PSMA tracers, is excreted in urine as opposed to the mainly hepatobiliary excretion of [^18^F]PSMA-1007. Theoretically this is an advantage for [^18^F]PSMA-1007 in T-staging since the prostate is adjacent to the urinary bladder. The lower performance in our study could be due to differences in patient selection, the difference in clinical experience and practice of readers and the low reliability of the measurement. A recent study by Tang et al. using [^18^F]PSMA-1007 evaluated, among other things, EPE and SVI in 130 patients. EPE criteria were “angulated contour of the prostate gland or obliteration of the rectoprostatic angle”, SVI criteria were not defined. The results were sensitivities and specificities of 0.23/1.0 and 0.52/1.0 respectively (Tang et al. 2023). Most notably, the previously mentioned study with 600 patients by Donswijk et al. compared four tracers, including [^18^F]PSMA-1007, and found no differences in diagnostic performance in T-staging (Donswijk et al. 2024).

### Interrater reliability and generalizability

To our knowledge, this is the first study using semi-standardized techniques for both quantitative (LCC) and qualitative (visual) aspects of T3 staging with PSMA PET/CT. The IRR for these techniques were moderate to substantial with ICC and k between 0.50 and 0.70, highest for LCC (ICC = 0.68). Donswijk et al. found moderate IRR for their visual evaluation with k between 0.40 and 0.50 (Donswijk et al. 2024). Sonni et al. found poor IRR for a visual interpretation of EPE and SVI (ICC 0.20 and 0.08 respectively) (Sonni et al. 2022).

In PSMA PET/CT the indicators of EPE and SVI are the direct visualization of tracer uptake outside the prostate or in the vesicles and, for EPE, the indirect measure of LCC. Both these indicators are dependent on SUV window levels. The challenge is similar to that of tumor volume determination in radiotherapy. In that setting a fixed threshold of 42% of SUVmax is often used although no optimal threshold has been established. Thresholds of 30–70% have been proposed, mostly based on studies of FDG PET. With small lesions (such as in prostate cancer), low thresholds should be avoided (Tamal 2020). The standardized NEMA Hot metal blue LUT we used switches from deep blue to reddish in the range of about 47–55% which serves as the visual tumor delineation zone. The exact threshold used is likely somewhat arbitrary but a LUT with narrower visual threshold, or even a stepped color palette such as the NEMA PET 20 step, could improve the reliability of the test.

Another factor that could reduce reliability is the subjectivity involved in choosing when to measure SUVmax close to the capsule in heterogenous lesions (as described in Methods). Both in this choice and in the visual interpretations the subjective component might be reduced with training materials.

We used 5 mm axial CT slices which are not optimal for non-axial reconstructions and could affect measurements in sagittal and coronal planes. Many PSMA PET scans are performed with a low-dose CT. For eventual clinical implementation of LCC measurements, optimization of protocols and reconstructions should be pursued.

To further improve reliability and to overcome the heterogeneity of study data standardization of PET interpretation is required, such as has been accomplished for MRI with PI-RADS (Weinreb et al. 2016). The Swedish prostate cancer guidelines apply a Likert scale to EPE and SVI evaluation with MRI (Table 2). It is based on ESUR guidelines but with clearly defined criteria for the EPE scale, including LCC, and might serve as a model for PET standardization (Regionala cancercentrum 2024).

### Limitations

The retrospective nature of this study introduces a selection bias. Patients with strong suspicion of T-stage 3 or higher are more likely to be selected for radiotherapy rather than prostatectomy, or may not be eligible for curative therapy. This bias could lower the diagnostic performance of PET and MRI, mainly the sensitivity.

For the MRI scans a variety of protocols and scanners were used, reflecting a real world setting but possibly making interpretation more difficult compared to the PET/CT scans where only one type of scanner was used.

## Conclusion

In this retrospective study, we compared semi-standardized quantitative and qualitative aspects of [^18^F]PSMA-1007 PET/CT to MRI in the evaluation of T3 (EPE and SVI) staging in prostate cancer. For EPE with PSMA PET/CT the quantitative length of capsule contact measurement performed best with high specificity, moderate accuracy, and substantial agreement between readers. The qualitative visual PSMA PET/CT evaluation of EPE performed relatively poorly with low accuracy and moderate agreement. MRI also achieved moderate accuracy with higher sensitivity than PET. For SVI both modalities had high specificity and low sensitivity, but the prevalence of SVI was low.

In this and other studies PSMA PET has shown promise in T3 staging of prostate cancer, but with low reproducibility between studies. To accurately assess the diagnostic performance of PSMA PET further standardization of interpretation is needed. The measurement of capsular contact may outperform the visual assessment of EPE in PSMA PET/CT and should be investigated further.

## Supplementary Information

Supplementary Material 1.

Supplementary Material 2.

## Data Availability

Data from the study are available from the corresponding author on reasonable request.

## Abbreviations

ADC: Apparent diffusion coefficient
AUC: Area under the curve
CT: Computed tomography
EPE: Extraprostatic extension
ICC: Intraclass correlation coefficient
IRR: Interrater reliability
LCC: Length of capsular contact
LUT: Look up table
NEMA: National Electrical Manufacturers Association
PET: Positron emission tomography
PI-RADS: Prostate Imaging–Reporting and Data System
PSA: Prostate specific antigen
PSMA: Prostate Specific
ROC: Receiver operating characteristic
SV: Seminal vesicle
SVI: Seminal vesicle invasion
SUV: Standardized uptake value
TNM: Tumor node metastasis
