# Supplementary material for: Semi-standardized evaluation of extraprostatic extension and seminal vesicle invasion with [18F]PSMA-1007 PET/CT: a comparison to MRI using histopathology as reference
Source: EJNMMI Rep. 2025 Jan 3;9(1):1. doi: 10.1186/s41824-024-00234-4 (PMC11695508; doi:10.1186/s41824-024-00234-4)
Supplement: Supplementary file 1 — Supplementary Material 1. [file 41824_2024_234_MOESM1_ESM.pdf]

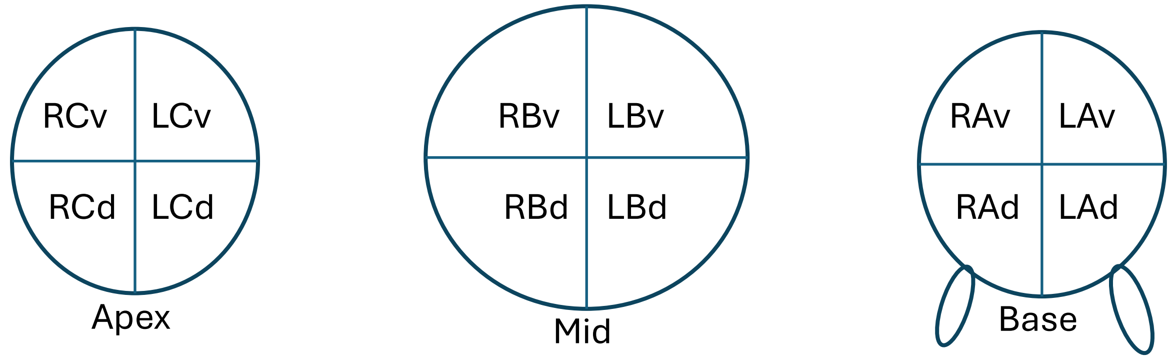

A: Division of prostate. Directly neigbouring sections of RCv are LCv, RCd, RBv, LBv and RBd

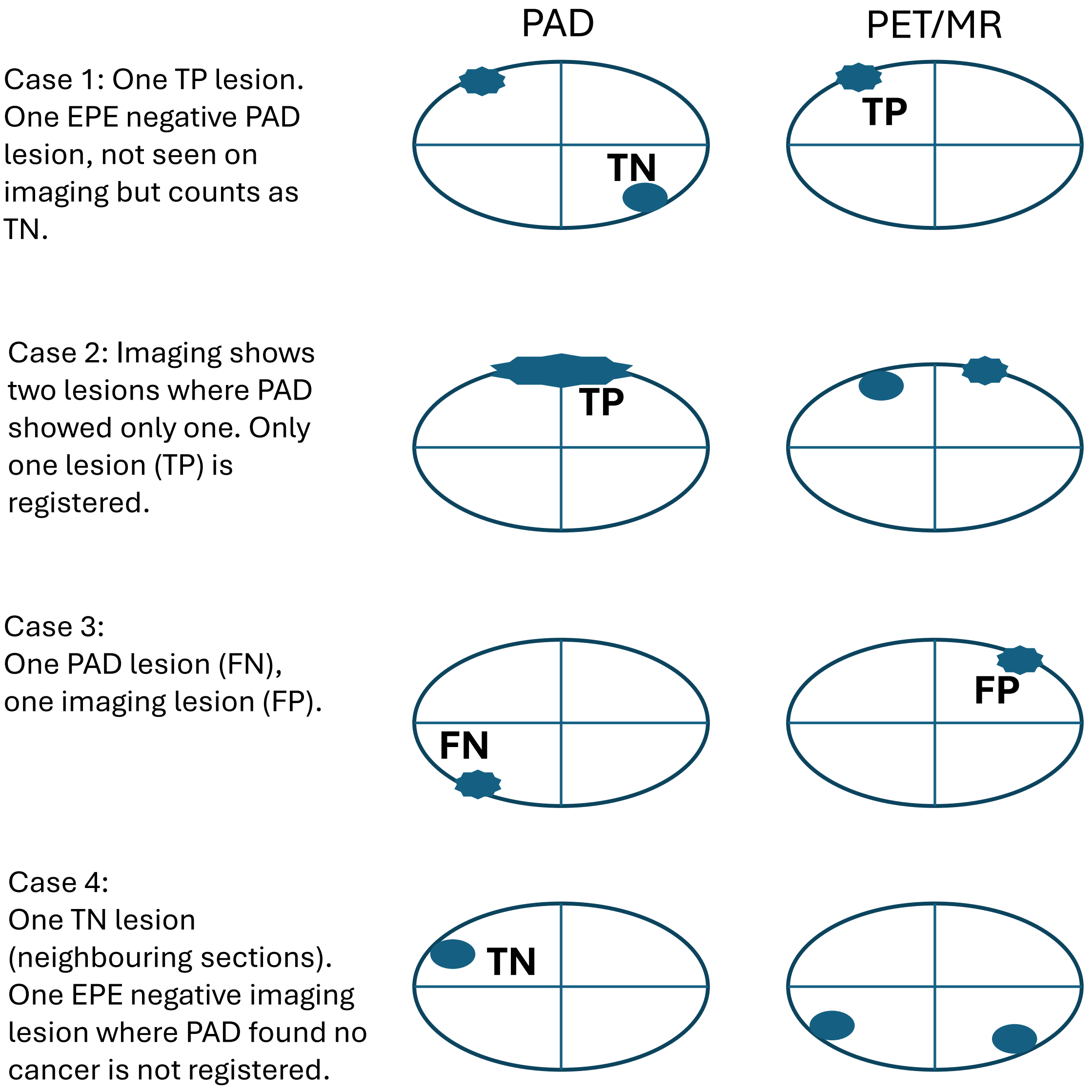

B: Schematic examples of comparisons on a lesion level in four hypothetical cases.
