# Supplementary material for: Semi-standardized evaluation of extraprostatic extension and seminal vesicle invasion with [18F]PSMA-1007 PET/CT: a comparison to MRI using histopathology as reference
Source: EJNMMI Rep. 2025 Jan 3;9(1):1. doi: 10.1186/s41824-024-00234-4 (PMC11695508; doi:10.1186/s41824-024-00234-4)
Supplement: Supplementary file 2 — Supplementary Material 2. [file 41824_2024_234_MOESM2_ESM.pdf]

Analysis	Sensitivity	Specificity	PPV	NPV	Accuracy	TP/FP/FN/TN
**EPE on a patient basis (n=124)**						
PET C^{3+}	.70 (.57–.82)	.49 (.38–.60)	.48 (.37–.59)	.71 (.57–.82)	.57 (.49–.66)	35/38/15/36
PET C^{3-}	.68 (.54–.80)	.54 (.32–.65)	.50 (.38–.62)	.71 (.59–.82)	.60 (.51–.68)	34/34/16/40
PET $C^{3-,\ LCC13}$	.60 (.46.–73)	.73 (.62–.82)	.60 (.46–.73)	.73 (.62–.82)	.68 (.60–.76)	30/20/20/54
PET $C^{3-,\ LCC14}$	.54 (.40–.67)	.76 (.65–.85)	.60 (.45–.74)	.71 (.60–.80)	.67 (.59–.75)	27/18/23/56
PET V^{3+}	.38 (.25–.52)	.73 (.62–.82)	.49 (.34–.64)	.64 (.53–.73)	.59 (.50–.68)	19/20/31/54
PET V^{3-}	.28 (.17–.41)	.82 (.73–.90)	.52 (.34–.70)	.63 (.53–.72)	.60 (.52–.69)	14/13/36/61
PET LCC	.64 (.50–.76)	.60 (.48–.70)	.52 (.39–.64)	.71 (.59–.81)	.61 (.53–.70)	32/30/18/44
PET LCC^{LCC13}	.54 (.40–.67)	.84 (.74-.91)	.69 (.54–.82)	.73 (.63–.82)	.72 (.64–.80)	27/12/23/62
PET LCC^{LCC14}	.46 (.33–.60)	.91 (.83–.96)	.77 (.60–.89)	.71 (.62–.80)	.73 (.65–.80)	23/7/27/67
MR^{3+}	.90 (.80–.96)	.34 (.24–.45)	.48 (.38–.58)	.83 (.68–.94)	.56 (.48–.65)	45/49/5/25
MR^{3-}	.80 (.68–.89)	.64 (.52–.74)	.60 (.48–.71)	.83 (.71–.91)	.70 (.62–.78)	40/27/10/47
Removing patients where no lesions were found (PET n=108, MR n=121)						
PET C^{3+}	.78 (.65–.89)	.42 (.30–.54)	.50 (.39–.61)	.72 (.56–.85)	.57 (.48–.67)	36/36/10/26
PET C^{3-}	.76 (.63–.87)	.48 (.36–.61)	.52 (.40–.64)	.73 (.59–.85)	.60 (.51–.69)	35/32/11/30
MR^{3+}	.90 (.80–.96)	.31 (.21–.42)	.48 (.38–.58)	.82 (.64–.93)	.55 (.47–.64)	45/49/5/22
MR^{3-}	.80 (.68–.89)	.62 (.50–.73)	.60 (.48–.71)	.82 (.70–.90)	.69 (.61–.78)	40/27/10/44
**SVI on a patient basis (n=124)**						
PET^{3+}	.14 (.03–.38)	.99 (.96–1.0)	.67 (.16–.98)	.90 (.84–.95)	.90 (.84–.95)	2/1/12/109
PET^{3-}	.14 (.03–.38)	1.0 (.97–1.0)	1.0 (.16–1.0)	.90 (.84–.95)	.90 (.85–.96)	2/0/12/110
MR^{3+}	.64 (.38–.85)	.80 (.72–.87)	.29 (.15–.46)	.95 (.89–.98)	.78 (.71–.85)	9/22/5/88
MR^{3-}	.50 (.26–.75)	.92 (.86–.96)	.44 (.22–.68)	.94 (.88–.97)	.87 (.81–.93)	7/9/7/101
**EPE on a lesion basis (PET n=169, MRI n=168)**						
PET C^{3+}	.70 (.57–.82)	.66 (.58–.75)	.47 (.36–.58)	.84 (.76–.91)	.67 (.60–.75)	35/40/15/79
PET C^{3-}	.68 (.54–.80)	.71 (.62–.78)	.49 (.38–.61)	.84 (.76–.90)	.70 (.63–.77)	34/35/16/84
PET V^{3+}	.38 (.25–.52)	.82 (.75–.89)	.48 (.33–.63)	.76 (.68–.83)	.69 (.62–.76)	19/21/31/98
PET V^{3-}	.28 (.17–.41)	.88 (.82–.93)	.50 (.32–.68)	.75 (.67–.81)	.70 (.64–.77)	14/14/36/105
PET LCC	.64 (.50–.76)	.75 (.67–.82)	.52 (.39–.64)	.83 (.75–.89)	.72 (.65–.78)	32/30/18/89
MR^{3+}	.90 (.79–.96)	.55 (.46–.63)	.45 (.35–.55)	.93 (.85–.97)	.65 (.58–.72)	44/54/5/65
MR^{3-}	.78 (.65–.88)	.75 (.67–.82)	.56 (.44–.67)	.89 (.82–.94)	.76 (.69–.82)	38/30/11/89
**SVI on a lesion basis (n=248)**						
PET^{3+}	.18 (.05–.40)	1.0 (.98–1.0)	.75 (.28–.98)	.94 (.91–.97)	.94 (.91–.97)	3/1/14/230
PET^{3-}	.12 (.02–.32	1.0 (.98–1.0)	1.0 (.16–1.0)	.94 (.91–.96)	.94 (.91–.97)	2/0/15/231
MR^{3+}	.71 (.47–.88)	.89 (.84–.92)	.32 (.18–.47)	.98 (.95–.99)	.88 (.83–.92)	12/26/5/205
MR^{3-}	.47 (.25–.70)	.96 (.93–.98)	.44 (.23–.67)	.96 (.93–.98)	.92 (.89–.96)	8/10/9/221

C Combination of visual interpretation and LCC, cut-off ≥10 unless otherwise noted

V Only visual interpretation

LCC Only LCC, cut-off ≥10 unless otherwise noted

$^{3+}$ Likert 3 positive

$^{3-}$ Likert 3 negative

^{LCC13} LCC cut-off ≥13

^{LCC14} LCC cut-off ≥14
